# Hedonic Judgments of Chemical Compounds Are Correlated with Molecular Size

**DOI:** 10.3390/s110403667

**Published:** 2011-03-25

**Authors:** Manuel Zarzo

**Affiliations:** Department of Applied Statistics, Universidad Politécnica de Valencia, Camino de Vera s/n, 46022 Valencia, Spain; E-Mail: mazarcas@eio.upv.es; Tel.: +34-963877490; Fax: +34-963877499

**Keywords:** VOC, olfaction, pleasantness, hedonic valence, odor character descriptor, numerical odor profile

## Abstract

Different psychophysical works have reported that, when a wide range of odors is assessed, the hedonic dimension is the most salient. Hence, pleasantness is the most basic attribute of odor perception. Recent studies suggest that the molecular size of a given odorant is positively correlated with its hedonic character. This correlation was confirmed in the present study, but further basic molecular features affecting pleasantness were identified by means of multiple linear regression for the compounds contained in five chemical sets. For three of them, hedonic judgments are available in the literature. For a further two chemical sets, hedonic scores were estimated from odor character descriptions based on numerical profiles. Generally speaking, fairly similar equations were obtained for the prediction of hedonic judgments in the five chemical sets, with R^2^ values ranging from 0.46 to 0.71. The results suggest that larger molecules containing oxygen are more likely to be perceived as pleasant, while the opposite applies to carboxylic acids and sulfur compounds.

## 1. Introduction

Electronic noses are comprised of an array of non-specific chemical sensors that provide a characteristic response pattern for odorous samples. With proper calibration, these devices can be used to assess odor intensity [1] and perform discrimination tasks. Plenty of literature is available on this subject (for a review, see [2]), but the prediction of odor character according to molecular structure is still a challenge. Although olfactory perception space is highly dimensional due to the large number of different olfactory receptors involved in odorant recognition [3], it is widely accepted that pleasantness is the most salient dimension when a wide range of smells is assessed at a similar odor intensity. Evaluation of whether one likes or dislikes an odor is known as hedonic valence (from the Ancient Greek: hçdonç = pleasure). The idea that odors could be classified in three main categories: pleasant, intermediate and unpleasant, was first proposed long ago [4,5].

In a recent study, the pleasantness of 76 odorants was rated by human appraisers as well as by an electronic nose. A significant correlation was found comparing the hedonic estimations from the electronic nose, calibrated with the 76 initial odorants, as compared with the human hedonic judgments of 21 odorants (*r* = 0.45, *p* < 0.0001) and 22 essential oils (*r* = 0.64, *p* < 0.0001) [6]. Similar works have also used electronic noses to predict hedonic assessments of various odorous samples [7,8].

Although the prediction of the hedonic tone of aroma chemicals by means of electronic noses has not yet been given much attention, recent studies suggest a link between odorant pleasantness and molecular structure. These studies are reviewed below, as well as different psychophysical olfactory studies supporting the hypothesis that pleasantness is the most basic attribute for the classification of odors.

### 1.1. The Hedonic Dimension of Odor Perception

One procedure for characterizing the smell of a set of odorants is to assess the similarity of all pairwise combinations of samples using a numerical scale (e.g., zero if the smell is completely different, up to 9 if it is almost identical). The resulting data structure is a symmetrical matrix that can be analyzed using multidimensional scaling (MDS). This method was applied by Yoshida [9], who selected 20 pure chemicals and asked a panel of five naive subjects to rate the odor similarity of all possible pairs of compounds. The first factor of the MDS solution was interpreted as a hedonic dimension, and the second factor as a sweet/pungent dimension.

Using a panel of 20 subjects, Woskow [10] obtained odor similarities for a set of 25 odorants and analyzed the data with MDS. Two dimensions were identified: one intensive (weak or strong odor sensation) and one hedonic. Davis [11] analyzed the same data using different techniques, and similar conclusions were drawn. These results suggest that odor strength will probably be the first attribute to emerge if someone is asked to describe the most dissimilar aspect of different odorants, unless they are all assessed at a similar odor intensity. Berglund and coworkers applied MDS to the similarities of odor quality comparison of 21 chemical compounds, and pleasantness came forward as the most salient dimension of olfactory perception [12].

In another study, 20 students assessed the odor of 40 essential oils that were selected to cover a broad spectrum of perfumery odors [13]. Panelists were asked to rate the similarity of each sample according to 32 reference test odorants on a 0–8 scale. A principal components analysis (PCA) performed on the average ratings yielded seven factors. The first one accounted for the average profile, revealing that the most unpleasant reference odors were rated with a lower frequency. The second component was related to the hedonic dimension. Similar results appeared using MDS [13].

Schiffman and coworkers [14] asked a panel of 12 subjects to smell 19 chemicals and rate the similarity of odor character for all possible pairs of odorants. Each odorant was also scored according to 22 semantic differential scales. The first factor of the MDS analysis was related to pleasantness and discriminated odorants described as fragrant and good from those regarded as foul, bad, and putrid. The second factor was determined by the descriptors “sharp” and “burning”, and was interpreted as a “tactile” dimension. A similar two-dimensional space was obtained in a previous study [15,16].

Coxon and coworkers [17] obtained numerical odor profiles for 23 compounds according to nine relevant odor descriptors. Each compound was rated on a 0–10 scale based on how it exemplified each of the nine selected descriptors. An MDS analysis yielded a four-dimensional solution, and the first dimension was related to hedonic aspects. In a similar study, 37 aroma chemicals were rated on a 7-point scale according to 55 descriptive characteristics, and the first principal component (PC1) was interpreted as pleasantness [8].

Stevens and O’Connell [18] asked a panel of 42 volunteers to smell a set of 15 odorants matched for intensity and to sort them into groups of samples with a similar odor. Next, pairwise similarity estimates between odors were derived by counting the number of times that two odors were sorted into the same group, which led to a co-occurrence matrix suitable for MDS analysis. In a similar experiment, three panels conducted a sorting task with 40 odorants [19]. In both studies, the first dimension of the MDS solution discriminated the most unpleasant odorants.

This odor sorting methodology, first proposed by Lawless [20], was also used by Sicard and coworkers [21], who asked a group of 40 subjects to assess 20 odorants and to group them according to odor resemblance. The results led to a co-occurrence matrix that was analyzed using factorial correspondence analysis. The first factor discriminated three odorants that were described as unpleasant. Dubois [22] conducted an experiment on a set of 16 familiar odorants. Two panels of 40 subjects were instructed to sort the odorants freely. Four classes of odorants were identified, and the most unpleasant odors were clustered together.

In another reported experiment, three panels of 30 students assessed 40 samples representative of familiar odors and rated odor character on a numerical scale according to 11 categories [23]. In a recent analysis of these numerical odor profiles, PC1 was interpreted as the hedonic dimension, and PC2 basically discriminated between food *versus* non-food odors [24].

### 1.2. Relationship of Hedonic Perception and Molecular Size

The Atlas of Odor Character Profiles [25] contains numerical olfactory descriptions for 144 monomolecular compounds and 16 additional samples. From this database, usually referred to as Dravnieks’ Atlas, nine odorants were selected in a recent study and the pairwise distance between two odorants along PC1 was found to be correlated with the pairwise distance in odorant pleasantness perceived by a panel [26]. Based on the results, PC1 was interpreted as the hedonic dimension. Hence, the projections of compounds along the direction determined by PC1 could be interpreted as estimated scores of pleasantness. In the same work, 1,513 physicochemical molecular descriptors were generated for 1,565 odorants. The 144 chemicals of the Atlas were projected over PC1 of this physicochemical database, and a significant correlation (*r* = 0.49, *p* < 0.001) was found between these projections and the scores for pleasantness. Similar results were obtained in a confirmatory experiment. Based on the correlation observed, the authors suggested that the perception of pleasantness (*i.e.*, the primary axis of olfactory perception) reflects the major axis of physicochemical properties. One of the variables with highest loading in PC1 was the number of non-hydrogen atoms, which accounts for molecular size [26].

The present work further investigates the correlation between the number of non-hydrogen atoms and hedonic judgments deduced from Dravnieks’ Atlas. Olfactory data from four additional psychophysical studies reported in the literature was also analyzed, and a procedure for estimating hedonic scores from odor character profiles was proposed. This issue is of relevant interest because recent studies further support a relationship between hedonic perception of odorants and their molecular structure [6,27–31], which suggests that hedonic perception is partly innate, in contrast to the more common view that hedonic aspects are predominantly shaped by experience.

## 2. Materials and Methods

### 2.1. Hedonic Scores from the Literature

Different research works have reported hedonic ratings of odorants. Harper and coworkers [32] asked different sensory panels to smell 53 monomolecular odorants and describe the odor character as well as the hedonic tone. Based on the hedonic ratings, the authors labeled each compound on a semantic scale from very unpleasant to very pleasant. I coded these hedonic descriptions on a numerical scale as follows: −4 (very unpleasant), −2.67 (moderately unpleasant), −1.33 (slightly unpleasant), 0 (neutral, *i.e.*, neither pleasant nor unpleasant), 1.33 (slightly pleasant), 2.67 (moderately pleasant), and 4 (very pleasant). Two chemicals were basically odorless and were disregarded. The variable containing the hedonic scores rated by the panel for the 51 chemical odorants was called H_Harper_.

In the first experiment conducted by Harper *et al.* [32], a panel of inexperienced subjects assessed the odor character of the 53 compounds. Odor description was essentially spontaneous and most subjects used only one word. Odor profiles were obtained by the authors in terms of the percentage of subjects using particular descriptors. For example, in the case of benzaldehyde, 84% of respondents described it as “almond.” Thus, the odor description of this compound can be regarded as a numerical profile containing a value of 84 for “almond”, and zero for the other terms. According to this criterion, a matrix was arranged containing 51 observations (odorants, in rows) by 57 variables (odor descriptors, in columns), which was called Harper’s matrix. A given element of this matrix, x_ij_, indicates the percentage of subjects in the panel that labeled the *i*-th chemical with the *j*-th descriptor. This method applied here to transform semantic odor descriptions directly obtained from a panel into numerical odor profiles was proposed by Dravnieks *et al.* [33] and later used in the compilation of Dravnieks’ Atlas [25]. This Atlas actually also contains the percentage of panelists who used each descriptor for each sample, which is correlated with the average score obtained from the panel.

Six of the 57 descriptors in Harper’s matrix (“estery”, “cough mixture”, “methylated spirits”, “*p*-dichlorobenzene”, “pyridine”, and “formalin”) are not included in Dravnieks’ comprehensive list of 146 terms and were discarded. “Wintergreen” was assumed to be equivalent to “medicinal” because they are related odors [24]. For the remaining 51 descriptors, the hedonic tones are available from the work of Dravnieks *et al.* [34]. In the reported study, a panel of about 120 individuals rated the hedonic tone of 146 odor descriptors on a numerical scale ranging from −4 for the most unpleasant to +4 for the most pleasant. These hedonic tones, which will be called HT_D84_, are useful to estimate the hedonic score of a given odorant when only the odor character profile is known. The procedure applied for estimating hedonic scores from Harper’s matrix is explained for benzyl acetate as an example. This odorant was described by 36% of panelists as “fruity” and by 8% of panelists as “sweet.” Taking into account the hedonic tone of both descriptors (2.23 for “fruity”; 2.03 for “sweet”), the hedonic score of this chemical was calculated as the weighted average of HT_D84_ according to the odor profile as: 2.23·(36/44) + 2.03·(8/44). Hence, the hedonic score of the *i*-th chemical in Harper’s matrix was estimated according to Equation (1):

(1) $$H_{i}=\sum_{j=1}^{J}\left({\mathrm{HT}}_{{D84}_{j}}\cdot \frac{x_{\mathrm{ij}}}{x_{i,1}+x_{i,2}+\ldots +x_{i,J}}\right)$$

HT_D84*_j_*_ being the hedonic tone of the *j*-th descriptor in the matrix, and *J* the number of columns (*i.e.*, 51 in this case). The resulting values are expressed in a −4 to 4 scale, as is the case of HT_D84_. Yeshurun and Sobel [29] claim that there is an almost unique pleasantness score to each odor object, which supports the procedure used here. The correlation between hedonic scores obtained by sensory analysis, H_Harper_, and the ones resulting from Equation (1) was studied.

Hedonic information of chemical compounds is also available from the work of Moncrieff [35], who obtained hedonic rankings for 132 odorant materials by means of a panel of 12 individuals. Each panelist assigned a preference ranking for each odorous sample. Next, the rankings assigned by panelists were averaged for each odorant. These preference rankings obtained by Moncrieff will be called PR_M_.

**Figure 1.**
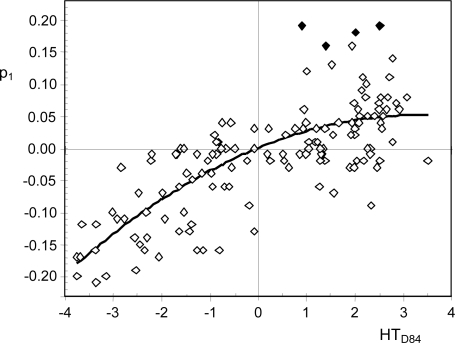
Plot of fitted regression analysis of p_1_ (loadings in the formation of the first principal component of Dravnieks’ Atlas) *versus* HT_D84_ (hedonic tones proposed by [34]). The fitted curve corresponds to Equation (4). The four highest residuals (filled points) are moderate outliers.

Wright and Michels [36] asked a group of 8 4 subjects to rate 50 odorous samples (45 monomolecular compounds and five replicates) on a 6-point scale according to their similarity to each of nine odorant standards which covered a wide range of olfactory quality. The 50 odorants were then correlated with each other across the nine standards. The resulting 50 × 50 correlation matrix was studied using factor analysis methods, and eight factors were obtained. The same matrix was re-analyzed by Schiffman [15,16] using MDS. A two-dimensional map was obtained, showing that the first dimension discriminated two clusters of compounds, one being more pleasant than the other. The projections of the 45 chemicals over the first dimension can therefore be regarded as hedonic scores. These values were obtained from Figure 3 of Schiffman [15], which is equivalent to Figure 1 of Schiffman [16], and then these were multiplied by a constant to express them in a scale from −4 to 4. The resulting variable was called H_W&M_.

### 2.2. Hedonic Scores from Dravnieks’ Atlas

Dravnieks’ Atlas contains numerical odor descriptions for 160 samples, that were assessed at a similar odor intensity by a panel of about 120 subjects. The panel was provided with a list of 146 commonly used odor descriptors. Panelists were requested to smell each sample and to score each descriptor on a numerical scale from 0 to 5, reflecting “absent” to “extremely” representative. For each odorant and each descriptor, the Atlas indicates the percentage applicability parameter. This ranges from 0 to 100 and was calculated according to the average score from the panel and the percentage of panelists who used the term. I arranged these data in a matrix containing 160 odorant samples (in rows) by 146 variables (odor character descriptors, in columns). A preliminary multivariate analysis of this matrix (unpublished data) suggested that percentage applicability values under 4 (*i.e.*, under 4% of the maximum limit) are basically random noise, and setting these to zero is therefore convenient. This threshold of significance was also established by Dravnieks [25], and a similar value was obtained in a reported analysis of odor profile databases [37]. Applying this procedure, six variables resulted with all null values and were excluded (“apple”, “laurel leaves”, “beery”, “rope”, “eggy”, and “soupy”). The values corresponding to “cheesy” were also excluded because these were found to be identical to “caraway” for all samples, which is nonsense. Equation (1) was applied to the resulting matrix with 139 variables in order to estimate hedonic scores for the 160 odorous samples, which were called H_At-eq1_. Reported evidence suggests that PC1 of Dravnieks’ Atlas can be interpreted as the hedonic dimension [26,38]. The projections of odorants over this direction, which are often called t_1_ scores, can therefore be regarded as an indirect estimation of hedonic ratings. In order to obtain these values, which were referred to as H_At-t1_, a PCA was carried out with Dravanieks’ matrix, using the SIMCA-P 10.0 software (www.umetrics.com). Variables were autoscaled (*i.e.*, mean-centered and scaled to unit variance) prior to the analysis, which is a common data pretreatment in PCA.

PC1 is the direction of maximum data variance obtained as a linear combination of the original variables. The contribution of variables to the formation of PC1 is called p_1_ loadings. The correlation between these loadings and the hedonic tones HT_D84_ of the corresponding reference descriptors was studied, as well as the correlation between H_At-eq1_ and H_At-t1_.

### 2.3. Hedonic Scores from the Amoore and Venstrom Database

Another olfactory database of chemical compounds was put forward by Amoore and Venstrom [39] (referred to hereafter as the A&V database). A panel of 29 members assessed 107 chemicals that had been equated for perceived odor intensity. Panelists smelled each sample and rated the smell similarity to seven standard odorants on a numerical scale from 0 to 8. Each standard was selected as a reference for a primary odor: “ethereal”, “camphoraceous”, “musky”, “floral”, “minty”, “pungent”, and “putrid”. Data from the original publication [39] was arranged in a matrix containing 107 observations by seven variables. The elements of this matrix, x_ij_, represent the similarity of odorant *i* compared with the *j*-th odor reference, according to the panel. Taking into account that hedonic tone HT_D84_ is known for these seven descriptors, Equation (1) was applied to estimate hedonic scores, which were called H_A&V-eq1_.

### 2.4. Correlation between Hedonic Scores and Molecular Size

According to the methodology described above, hedonic scores were obtained for five sets of chemical stimuli: (i) the set of 51 odorous compounds used by Harper *et al.* [32], (ii) chemical odorants used by Moncrieff [35], (iii) the 45 compounds used by Wright and Michels [36], (iv) chemicals contained in Dravnieks’ Atlas [25], and (v) the 107 compounds in the A&V database [39].

Among the 132 odorant materials used by Moncrieff [35], there are 68 monomolecular chemicals. Seven isomers were excluded because they contain information which is redundant for the purpose of this work. The remaining 61 chemicals were regarded as the second chemical set.

Dravnieks’ Atlas comprises 160 odorous samples: 144 single chemical compounds, eight natural oils, two simple mixtures, five complex mixtures, and one blank. Obviously, the comparison of hedonic character *versus* molecular size can only be made with the monomolecular compounds. Hence, all mixtures as well as the blank (dipropylene glycol), which was basically odorless, were disregarded. Moreover, six replicates of the chemical compounds that had been evaluated at a higher concentration were also excluded.

Among the natural materials included in the Atlas, the odor of five of them is basically determined by a major component (shown in brackets): eucalyptus oil (eucalyptol), garlic oil (allicin), onion oil (*n*-propyl disulfide), oenanthic ether (ethyl heptanoate), and patchouli oil (patchouli alcohol). The resulting group of 143 compounds (*i.e.*, 144 − 6 + 5) was considered to be the fourth chemical set.

The following variables describing basic molecular features were obtained for all chemicals in the five sets: molecular weight, total number of atoms, total number of atoms except hydrogen, number of carbon atoms, number of nitrogen atoms, and so on for each atom. Indicator variables providing information about the presence or absence of a particular type of atom were also considered. Additional indicator variables were used for carboxylic acids and amines. Next, multiple linear regression was applied in order to study the relationship between these molecular descriptors and the hedonic scores. Regression models were developed using the Statgraphics 5.1 software. In all cases, it was verified that residuals followed an approximately normal distribution and no outliers were identified.

### 2.5. Searching for the Hedonic Dimension in Two Odor Profile Databases

Given that the A&V database contains odor similarities to only seven standard odors that were assumed to account for independent dimensions of odor character [39], it is of interest to determine if pleasantness is also a salient dimension in this case. For this purpose, a PCA was carried out after applying the autoscaling pretreatment. The correlation between p_1_ loadings and the hedonic tones HT_D84_ of the corresponding reference descriptors was studied.

One of the largest databases of numerical odor profiles was obtained by Boelens and Haring [40]. A panel of six perfumers smelled 309 chemical compounds and rated the odor similarity to 30 standards on a scale from 0 to 9. This database was analyzed in recent studies [38,41], and the hedonic dimension did not show up clearly. The reason is somewhat uncertain, but one hypothesis is that this database basically contains pleasant odorants. In order to further investigate this issue, the hedonic scores of the 309 compounds were estimated using the Equations obtained from the other chemical sets.

## 3. Results

### 3.1. Hedonic Scores from the Literature and Estimated Values

In the experiment reported by Harper *et al.* [32], odorants were carefully chosen in order to provide as representative as possible a selection of all odors, ranging from very pleasant to very unpleasant. Actually, the average value of H_Harper_ is nearly zero (0.08). The linear relationship between H_Harper_ and the hedonic scores estimated from Equation (1), which were called H_Harp-eq1_, is indicated in Equation (2), resulting *r* = 0.824:

(2) $$H_{\text{Harper}}=0.26+1.03\cdot H_{\text{Harp}-\text{eq1}}$$

The slope is statistically significant (*p* < 0.0001), but not the constant (*p* = 0.092). If it is removed, the slope becomes 1.007 (*i.e.*, H_Harper_ ≈ H_Harp-eq1_) which is intuitively appealing, because both variables are expressed in the same scale from −4 to 4. Given this correspondence between H_Harper_ and H_Harp-eq1_, it was assumed that hedonic scores estimated by means of Equation (1) for the Atlas and the A&V database (*i.e.*, H_At-eq1_ and H_A&V-eq1_) are also directly comparable with H_Harper_ values.

Values of PR_M_ range from 5.1 to 121.3. Among the chemical compounds assessed by Moncrieff [35], 17 of them were verified as also having been used by Harper *et al.* [32]. For these compounds, the linear regression between H_Harper_ and PR_M_ is indicated in Equation (3). The correlation coefficient is negative (*r* = −0.726, *p* = 0.001) because lower rankings correspond to pleasant odors:

(3) $$H_{\text{Harper}}=7.11-0.091\cdot {\text{PR}}_{M}$$

PC1 of Dravnieks’ matrix accounts for 13.1% of the total data variability. The contributions of variables in the formation of PC1 (*i.e.*, p_1_ loadings) are correlated with the hedonic tones HT_D84_ (*r* = 0.74). By conducting a multiple lineal regression, it was found that the quadratic effect was also statistically significant (*p* = 0.014), but the constant was not (*p* = 0.30). Thus, a new model was fitted without the constant (Equation (4)), resulting R^2^ = 0.57 (Figure 1):

(4) $$p_{1}=0.0309\cdot {\text{HT}}_{\text{D84}}-0.0046\cdot {\left({\text{HT}}_{\text{D84}}\right)}^{2}$$

This quadratic Equation crosses the origin of coordinates and, hence, descriptors with p_1_ > 0 can be considered pleasant (HT_D84_ > 0) while the opposite applies to those with p_1_ < 0. Hence, p_1_ loadings can be regarded as unbiased estimations of the hedonic tone of descriptors. The four highest residuals (see Figure 1) come forward as moderate outliers and they interestingly correspond to the pleasant descriptors with highest average (*i.e.*, the ones most frequently scored): “light”, “sweet”, “aromatic”, and “fragrant.”

Hedonic scores H_At-eq1_, obtained by applying Equation (1), are strongly correlated (*r* = 0.93) with those calculated as the projection of observations over PC1, H_At-t1_. Nonetheless, Equation (5) describes this relationship better because the coefficient associated with the quadratic term is statistically significant (*p* < 0.0001), resulting R^2^ = 0.895:

(5) $$H_{\text{At}-t1}=-0.297+3.365\cdot H_{\text{At}-\text{eq}1}-0.491\cdot {\left(H_{\text{At}-\text{eq}1}\right)}^{2}$$

In this equation, the constant is not clearly significant (p = 0.060), which indicates that it could be removed from the model. This result suggests that both procedures for estimating hedonic scores are not biased, that is, if one method predicts a given odorant as unpleasant, the other one will do the same on average. The H_At-eq1_ variable ranges from −2.60 to 2.14, *i.e.*, with a similar range for positive and negative values. By contrast, H_At-t1_ ranges from −13.5 to 6.5, which means that the range for unpleasant odors is twice as large as for pleasant odors. Similarly, the range of p_1_ for unpleasant descriptors is also broader than the one for most pleasant descriptors, as deduced from the fitted model in Figure 1. For this reason, H_At-t1_ values were disregarded.

Among the 51 compounds in Harper’s matrix, 25 of them are also included in the Atlas. For these compounds, the linear relationship between H_Harper_ and H_At-eq1_ (Equation (6)) is statistically significant (*r* = 0.884, *p* < 0.0001), but not the constant (*p* = 0.34):

(6) $$H_{\text{Harper}}=0.185+1.228\cdot H_{\text{At}-\text{eq}1}$$

The slope does not differ significantly from unity (95% confidence interval, 0.95 to 1.51), which supports the assumption that H_At-eq1_ values are directly comparable with H_Harper_, as mentioned above. Equations (4), (5) and (6) confirm that the hedonic dimension is the most salient in Dravnieks’ Atlas.

### 3.2. Correlation between Hedonic Scores and Molecular Size

After obtaining the hedonic tone of compounds in the different databases, multiple linear regression was applied in order to study the relationship between perceived pleasantness and the basic molecular features.

Hedonic scores of the 51 odorants used by Harper *et al.* [32], H_Harper_, are correlated with molecular weight (*r* = 0.425, *p* = 0.002), but a higher correlation was obtained with the number of atoms except hydrogen, which will be referred to hereafter as N_at_ (*r* = 0.591, *p* < 0.0001). Interestingly, a similar result was obtained by Khan *et al.* [26], because PC1 of the physicochemical molecular data was correlated with hedonic character, and the number of non-hydrogen atoms was the eighth variable with highest loadings in PC1. After trying several models, the highest coefficient of determination (R^2^ = 0.537) was achieved with Equation (7):

(7) $$H_{\text{Harper}}=-2.56+0.23\cdot N_{\text{at}}+1.26\cdot I_{\text{ox}}-1.54\cdot I_{\text{sul}}$$

where I_ox_ is an indicator variable that takes a value of 1 if the molecule contains one or more atoms of oxygen, and zero otherwise. Similarly, I_sul_ indicates the presence of a sulfur atom. The effect of N_at_ is statistically significant (*p* = 0.0001) as well as I_ox_ (*p* = 0.008), but not so clearly in the case of I_sul_ (*p* = 0.050), probably because this chemical set only contains four sulfur compounds.

For the 61 chemicals used by Moncrieff [35], the best regression model that relates preference ratings and molecular features is Equation (8), resulting R^2^ = 0.707. All variables are statistically significant (p ≤ 0.003). Taking into account Equation (3) that relates H_Harper_ and PR_M_, Equation (8) turns into Equation (9), which is equivalent. Interestingly, the coefficients of Equations (7) and (9) are similar:

(8) $${\text{PR}}_{M}=106.9-2.49\cdot N_{\text{at}}-17.33\cdot I_{\text{ox}}+21.6\cdot I_{\text{sul}}+28.32\cdot I_{\text{acid}}+20.77\cdot I_{\text{amine}}$$

(9) $$H_{\text{Harper}}=-2.62+0.23\cdot N_{\text{at}}+1.58\cdot I_{\text{ox}}-1.96\cdot I_{\text{sul}}-2.58\cdot I_{\text{acid}}-1.89\cdot I_{\text{amine}}$$

For the 45 odorants used by Wright and Michels [36], Equation (10) relates hedonic scores and molecular features (R^2^ = 0.711). The indicator variable I_sul_ is moderately significant (*p* = 0.012) probably because there are only four sulfur compounds, but the other variables are clearly significant (p ≤ 0.003). Schiffman [15] also studied this chemical set and observed that carboxylic acids and sulfurs fell in the less pleasant space, as reflected by Equation (10):

(10) $$H_{W\&M}=-1.87+0.22\cdot N_{\text{at}}+2.60\cdot I_{\text{ox}}-2.23\cdot I_{\text{sul}}-3.51\cdot I_{\text{acid}}$$

The coefficients of N_at_ in Equations (7), (9) and (10) are very similar, but the one associated with I_ox_ is higher in Equation (10). The reason seems to be that none of the 12 unpleasant odorants in this chemical set contain oxygen, except the carboxylic acids. Hence, the model indicates that I_ox_ has a high predictive power of pleasantness, but the results from the other models suggest that a lower coefficient should be considered in order to estimate the hedonic score of any given molecule. This chemical set is somewhat different from the rest because odorants were perceived by the panel as clearly pleasant or unpleasant [15], and it does not contain chemicals with a neutral pleasantness. For this reason, I would not recommend Equation (10) for prediction. According to Moskowitz *et al.* [42], perceived pleasantness varies with concentration, and odors are described less frequently with a neutral valence if they are assessed at a higher intensity. But it is uncertain if this is the reason for the lack of neutral odorants in this case.

Data was fitted for the 143 chemicals in the Dravnieks’ Atlas according to Equation (11), resulting R^2^ = 0.503. A moderate significance was found for the quadratic term (*p* = 0.023), but the remaining variables are statistically significant (*p* ≤ 0.003). The interaction I_ox_·N_at_ modifies the coefficient associated with N_at_ for molecules containing oxygen. The largest molecule (N_at_ = 29) acts as an influential point in the model and it was excluded:

(11) $$H_{\text{At}-\text{eq1}}=-2.33+0.33\cdot N_{\text{at}}-0.0104\cdot N_{\text{at}}^{2}+0.057\cdot I_{\text{ox}}\cdot N_{\text{at}}-0.83\cdot I_{\text{sul}}-1.62\cdot I_{\text{acid}}$$

For the 107 odorants in the A&V database, the highest goodness-of-fit (R^2^ = 0.458) was obtained with Equation (12). All coefficients are statistically significant (*p* < 0.004):

(12) $$H_{A\&V-\text{eq1}}=-1.85+0.35\cdot N_{\text{at}}-0.014\cdot N_{\text{at}}{}^{2}+0.041\cdot I_{\text{ox}}\cdot N_{\text{at}}$$

Although the effect of I_sul_ and I_acid_ could not be studied in this case because this database only contains one sulfur compound and one carboxylic acid, it is interesting that Equations (11) and (12) are rather similar, because hedonic scores were obtained in both cases using Equation (1) and consequently both are expressed in the same scale.

### 3.3. Searching for the Hedonic Dimension in the A&V Database

The Amoore & Venstrom database [39] was analyzed with PCA in an attempt to identify correlation structures among the seven variables. One criterion usually applied is to focus on those PCs with an eigenvalue λ > 1. This condition is satisfied by PC1 (λ = 2.4), PC2 (λ = 1.6) and PC3 (λ = 1.2). Another criterion is based on the goodness-of-fit by cross-validation (Q^2^). In this case, Q^2^ is lower than the threshold value considered by the software (for PC1, Q^2^ = 0.07 < 0.13; for PC2, Q^2^ = −0.01 < 0.15). The results of PCA are usually easier to interpret if variables are normally distributed. Using a normal probability plot, it was observed that the seven variables follow a positive skewed distribution. In order to normalize the distribution, the logarithmic transformation was applied to three variables (“ethereal”, “musky” and “minty”), and the square-root transformation to the remaining ones. If a new PCA is carried out with the transformed variables, PC1 satisfies the cross-validation criterion (Q^2^ = 0.14 > 0.13) and accounts for 39.3% of the total data variance. This is not the case for PC2 (Q^2^ = 0.08 < 0.15), which explains 24.6% of the variance. Thus, PC1 can be considered as a relevant underlying dimension of the database.

**Figure 2.**
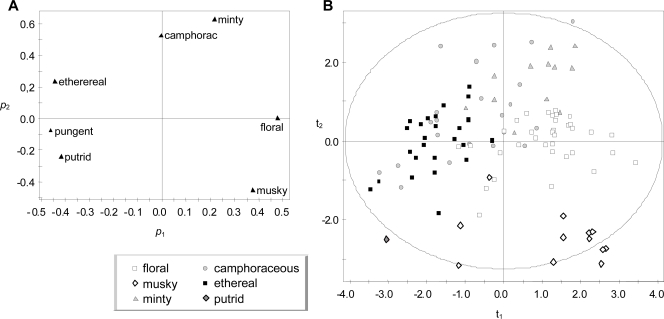
Results from the PCA performed with the database obtained by Amoore and Venstrom [39]. Prior to the analysis, variables were transformed for normality, and then mean-centered and scaled to unit variance. **(A)** Loading plot (p_2_ *vs.* p_1_) and **(B)** score plot (t_2_ *vs.* t_1_) for the first and second principal components. White squares represent the samples that were described with highest scores as floral, and so on, according to the caption (the pungent category does not appear because none of the samples was primarily described as pungent).

A scatter plot of the loadings corresponding to PC1 and PC2 [Figure 2(A)] highlights the similarities and dissimilarities among descriptors. “Minty” and “camphoraceous” present the highest loadings in PC2 and appear close to each other in this loading plot, but in a position opposite to “musky.” This observation suggests that minty and camphoraceous odors are related but clearly different from musky smells, which is consistent with other reported studies [43,44]. Hence, PC2 also provides relevant information, though it does not satisfy the cross-validation criterion of significance.

Hedonic tones HT_D84_ were assigned to the seven descriptors of the A&V database. The correlation between these values and p_1_ loadings is statistically significant (*r* = 0.88, *p* = 0.010). It might be argued that this observed significance level (*p*-value) is not low enough to interpret PC1 as the hedonic dimension because hedonic judgments are affected by many factors such as age, gender and personal experience (for review see [35,45]). In order to further investigate PC1, the projections of the 107 chemicals over this component (*i.e.*, t_1_ scores) were obtained. If these values are compared with the estimated hedonic scores H_A&V-eq1_, the correlation turns out to be statistically significant (*r* = 0.932, *p* < 0.0001). This p-value is much lower than in the previous case, which supports the interpretation of PC1 as the hedonic dimension. Taking into account this correlation, t_1_ scores can be interpreted as an indirect estimation of the hedonic character of the chemicals. However, if Equation (12) is modified using t_1_ as dependent variable instead of H_A&V-eq1_, a slightly lower goodness-of-fit (R^2^ = 0.432) is obtained.

It is worth noting that the hedonic dimension is even salient in the A&V database, though it contains just seven variables that were regarded by Amoore and Venstrom [39] as independent dimensions of odor character. This database was also analyzed by Wise *et al.* [46], who computed a dendogram that highlights the relationships between odorants. However, the results did not clearly reflect the hedonic dimension.

### 3.4. Hedonic Aspects of the Boelens and Haring Database

In the database obtained by Boelens and Haring [40], the average value of N_at_ is 13, which proves higher than in the other chemical sets studied here (Table 1). In order to estimate the hedonic scores of the 309 compounds in this database, it therefore seems more appropriate to apply Equation (11) or (12), given that both models take into account the quadratic effect of N_at_ observed for large molecules.

Applying Equation (11), the estimated hedonic tone is positive (*i.e.*, hedonic character predicted as pleasant) in 94.2% of the compounds, and a percentage of 98.4% was obtained using Equation (12). This result suggests that chemicals in this database are biased towards pleasant odors, which would explain why pleasantness was not found as a salient dimension in this database [38]. This hypothesis seems reasonable because odor profiles in the database of Boelens and Haring were obtained in the context of perfumery. The perfumer’s raw materials form a sample which is heavily biased towards pleasant items because the perfumer’s ultimate objective is, in general, a balanced product which is basically pleasant and appealing [47]. Nonetheless, some aroma chemicals may be unpleasant at higher concentrations.

## 4. Discussion

### 4.1. What Makes an Odorant Smell Pleasant or Unpleasant?

Although many psychophysical studies reviewed in the introduction have shown that the hedonic dimension is the most salient when a wide range of smells are assessed, a fundamental question still unsolved in the field of olfaction is what makes an odorant smell pleasant or unpleasant. One theory is that acquired semantic knowledge is one of the important factors that determines odor hedonic valence [35,45,48]. It is well established that hedonic odor perception is strongly influenced by odorant concentration [42], experience [35,45], learning [49], familiarity [50], culture [23,50], context, *etc.*

An alternative view derived from the work of Khan *et al.* [26] is that, in humans, the pleasantness of odors may partly be explained by the physicochemical properties of the odorant molecules themselves. A recent study has used Khan’s model to classify 20 odorants as pleasant or unpleasant, and a panel of human appraisers found that the perceived pleasantness of both groups was statistically different [30]. Thus, the olfactory system seems to be predisposed to discriminate environmental olfactory stimuli on the basis of their chemical structure. The fact that electronic noses are able to assess hedonic valence [6–8] is consistent with this hypothesis.

If hedonic odor value is indeed partly predetermined by odorant structure, then it could be hypothesized that other mammal species might have similar odor preferences to humans. Mandairon *et al.* [31] found a statistically significant correlation between odor investigation time in mice for 19 compounds and odor hedonic ratings in humans, which implies that the same odorants were similarly attractive to both species.

Studies with newborns also suggest that at least some aspects of olfactory pleasantness may be innate. Actually, human neonates (*i.e.*, with no exposure to culture or learning) are able to exhibit behavioral markers of repulsion in response to unpleasant odors [51]. Such predisposition in odor preference may be underlain by genetically programmed neural circuits, as has been suggested in the olfactory systems of mammals [52], and would explain why rodents bred for generations in predator-free laboratories are nevertheless averse to the smell of predators [53]. To sum up, experimental evidence supports the view that the hedonic perception of odorants is a complex process which involves both innate and learned components (for further discussion see [29]).

### 4.2. Effect of the Functional Group on Odor Character and Hedonic Perception

The fact that particular atoms or molecular features might affect the perceived hedonic character is not a new idea, because hedonic tones are associated with odor character descriptors [34], and chemists observed long ago that certain functional groups determine a specific odor character [54,55]. This issue was studied by Schafer & Brower [56], who found that a panel of 73 organic chemists were reasonably successful in identifying the functional groups of 36 unknown and unfamiliar odorants. Correct identifications of functional groups were made in 50–86% of the time for odorants containing amines, sulfur, esters, phenols, and carboxylic acids. The ability of human appraisers to distinguish between aliphatic odorants sharing the same number of carbon atoms but differing in their functional group has also been tested [57].

In the five chemical sets considered in the present study, the percentage of molecules containing oxygen is similar, about 73% (Table 1). The models obtained here indicate that the presence of oxygen (except in carboxylic acids) is likely to increase perceived pleasantness. This result is consistent with the fact that esters, ketones and lactones generally smell pleasant, which led to the suggestion that an oxygen linkage in a molecule is frequently associated with a pleasant odor [54].

Equations (7,9–11) are consistent with the well-known rule that molecules containing sulfur often have offensive smells [54]. These molecules generally smell “sulfidic”, which is an unpleasant descriptor (HT_D84_ = −2.45). Apart from sulfur, other particular atoms such as selenium, tellurium, phosphorus, bismuth, or arsenic are also likely to give an unpleasant odor [35]. Fourteen of the 107 compounds in the A&V database contain chlorine, which is a number high enough to study the effect of chlorine in hedonic character. However, this effect was not statistically significant (*p* = 0.66). A similar result (*p* = 0.65) was obtained in Moncrieff’s chemical set, which contains four chlorine compounds.

Carboxylic acids smell pungent and consequently tend to be described as unpleasant, as reflected by Equations (9–11). This effect was not identified in Harper’s nor in the A&V chemical sets because they contain too few carboxylic acids (Table 1). Actually, the descriptor “sour, vinegar” is unpleasant (HT_D84_ = −1.26) as well as “sharp, pungent, acid” (HT_D84_ = −2.34). The latter basically describes trigeminal sensation, which strongly depends on concentration. Nevertheless, many carboxylic acids, including medium to longer-chain acids, can smell unpleasant at sub-trigreminal concentrations (may smell cheesy or like body odor, for example).

It is also well known by chemists that amines produce a fishy-urinous odor, which results somewhat unpleasant (“ammonia”, HT_D84_ = −2.47; “fishy”, HT_D84_ = −1.98; “urinous”, HT_D84_ = −3.34). The negative effect of the indicator variable I_amine_ was statistically significant (*p* = 0.003) in Moncrieff’s chemical set (Equation (9)), which contains five amines, but not in the case of Dravnieks’ Atlas (*p* = 0.15). The remaining chemical sets do not contain enough amines to study this effect (Table 1). Apart from amines, no significant effect was found for the presence of nitrogen in the molecule. Further research will be required with a higher number of nitrogen compounds.

### 4.3. Correlation between Hedonic Scores and Molecular Size

Although recent experimental evidence supports the idea that odor pleasantness may partly be explained by odorant structure [6,27–31], the relationship between hedonic perception and molecular size has not been studied in depth. Khan *et al.* [26] found that the hedonic dimension was correlated with PC1 of the physicochemical molecular data, which accounted for 32% of the total variance. One of the variables with highest loading in PC1 was the number of non-hydrogen atoms, which suggests that this dimension could be basically interpreted as molecular size. Similar results were obtained by Schiffman [15,16], who calculated different molecular properties for the 45 compounds used by Wright and Michels [36] and found that molecular weight was the variable that best explained the discrimination of pleasant *versus* unpleasant odorants.

The results reported here confirm the positive correlation between N_at_ and hedonic character of a given molecule. Moreover, the coefficients of N_at_ in Equations (7), (9) and (10) are very similar. A quadratic effect of N_at_ was also observed, but only in the two chemical sets that contain a higher number of compounds (*i.e.*, the A&V and Dravnieks’ databases) and a higher average value of N_at_ (Table 1). This quadratic effect suggests that the hedonic character asymptotically tends towards a maximum value that is reached at N_at_ of about 14 [Equation (12)] or 19 [Equation (11)] for molecules containing oxygen (except carboxylic acids) but not sulfur.

Considering I_ox_ = 1 and other indicator variables equal to zero, Equations (7), (9), (11), and (12) become null for N_at_ = 5.7, 4.5, 7.5, and 5.9, respectively (average value, 5.9). A given molecule will thus be predicted as pleasant (*i.e.*, with a positive hedonic score) if it contains oxygen and at least 6 additional non-hydrogen atoms. Sulfur compounds or carboxylic acids are likely to be perceived as unpleasant. This rule should be regarded as a general trend found in this study, but many exceptions can be encountered. For example, many steroid-type molecules such as androstenone possess a urinous-sweaty odor, though the predictive models obtained here would classify them as pleasant odorants based on their high molecular size. On the other hand, it is a common observation that very subtle molecular changes can have profound shifts both in odor quality and hedonic valence.

The best predictive model obtained by Khan *et al.* [26] for the 144 chemicals in Dravnieks’ Atlas was based on seven PCs from the physicochemical data, and the correlation coefficient between PC perceptual hedonic values and predicted values from molecular structure was *r* = 0.59 (*p* < 0.0001). The same chemical set was studied here, and the correlation between hedonic values H_At-eq1_ and the ones predicted using Equation (11) was slightly higher (*r* = 0.71, *p* < 0.0001), although this Equation is based on very simple molecular features such as atom counts instead of complex dimensions of molecular descriptors. Models built with latent variables from a large matrix are difficult to interpret, and it is often advantageous to search for the best descriptor subset that improves the goodness-of-fit, as discussed in a latter study [27].

The main contribution of this paper is that a predictive model for hedonic tones was obtained with a similar goodness-of-fit to the model proposed by Khan *et al.* [26] despite using very simple molecular features. For this reason, better predictive models would probably have emerged if a detailed characterization of the molecular structure had been used by means of a large set of molecular descriptors, which encourages further studies aimed at understanding the relationship between molecular structure and hedonic odor character better. For this purpose, it would be necessary to use human assessors to obtain accurate hedonic ratings for a comprehensive set of compounds minimizing the effect of context, and the panelists’ culture and experience, which implies the use of large panels from different countries and cultures with participants of different ages, experience, *etc.* Moreover, given that odor intensity greatly affects pleasantness [42], it is also extremely important to assess all samples at the same odor intensity.

### 4.4. Role of the Hedonic Dimension of Odor Perception

Based on the finding that pleasantness is correlated with the most discriminating dimension of physicochemical molecular descriptors, Haddad *et al.* [28] suggested that, as with other senses, the olfactory system has evolved to exploit a fundamental regularity in the physical world. This hypothesis is appealing, but it still requires further investigation. The role of the hedonic dimension in olfaction was probably first discussed by Linnaeus [4], who suggested that fragrant and aromatic scents (*i.e.*, the most pleasant odors) are perceived to be *kindly and desirable to our nerves and even to life itself*, while unpleasant odors are those *repellent to life*. Similarly, Beebe-Center [58] considered that pleasant stimuli are often those that are beneficial to the body. *Anything that will tend to promote well-being, of the body or of the emotions, will also be pleasant: that what we need, we also like*. This rule especially applies for food scents: *what will be good for the body will usually be liked* [35]. Emotional effects elicited by odors and the role of olfaction in well-being have recently been discussed [59].

From an evolutionary standpoint, Proetz [60] proposed that odor qualities regarded as pleasant or unpleasant were at one time beneficial or harmful, respectively. Based on this idea, some authors have suggested that the high sensitivity of human olfaction in detecting hydrogen sulfide and amines is an evolutionary adaptation for detecting decaying food and toxic gases, which have been present for evolutionarily significant time periods in the atmosphere [1]. Amines and thiols are associated with harmful conditions derived from putrid food, and maybe for this reason they smell unpleasant. Thus, putrid fish produces trimethylamine, while the degradation of meat releases thiols and hydrogen sulfide, given that two amino acids contain sulfur [61]. The physical repulsion one experiences when smelling rotten meat is likely to be due to human evolutionary legacy: it might be an avoidance mechanism or an alarm signal telling us not to eat this. Although the odors themselves may not be toxic, their association with decaying material indicates something that is best avoided, as the decaying material itself can represent a health risk [62]. The smell of predators is generally perceived as unpleasant by mammals as an innate signal of danger [53]. In contrast to this, a pleasant smell would be a sign of beneficial conditions such as an edible food, a safe environment, or a fertile mate, and they all indeed generally smell pleasant to mammals [29]. The function of human olfaction as a warning signal to avoid environmental hazards has recently been reviewed [63].

## Figures and Tables

**Table 1.** Basic description of the chemical sets according to the empirical formula of the compounds.

**Chemical set**	**n^a^**	**N_at_^b^**			**Number of molecules containing^c^**					
		**min**	**max**	**average**	**oxygen**	**nitrogen**	**sulfur**	**chlorine**	**acid**	**amine**
Harper *et al.* [32]	51	1	21	8.2	35 (69%)	9	4	1	2	3
Moncrieff [35]	61	2	19	9.2	46 (75%)	9	5	4	3	5
Wright and Michels [36]	45	1	15	8.4	30 (67%)	4	4	2	5	0
Dravnieks’ Atlas [25]	143	4	29	10.7	112 (78%)	22	13	1	8	5
Amoore & Venstrom [39]	107	3	21	10.3	79 (74%)	7	1	14	1	0
Boelens and Haring [40]	309	3	22	13.0	287 (93%)	14	0	1	4	0

a Number of monomolecular compounds.

b Number of atoms in the molecule except hydrogen: minimum, maximum and average value.

c Number of molecules in the chemical set that contain an atom of oxygen, nitrogen, sulfur, chlorine, or molecules that are carboxylic acids or amines.
